# Translating Radiometric Requirements for Satellite Sensors to Match International Standards

**DOI:** 10.6028/jres.119.010

**Published:** 2014-07-14

**Authors:** Aaron Pearlman, Raju Datla, Raghu Kacker, Changyong Cao

**Affiliations:** 1 Earth Resources Technology, Inc., College Park, MD 20740; 2 National Institute of Standards and Technology, Gaithersburg, MD 20899; 3 NOAA/NESDIS/STAR, College Park, MD 20737

**Keywords:** error analysis, GOES-R Advanced Baseline Imager, Guide to the Expression of Uncertainty in Measurement (GUM), ISO standards, satellite sensor uncertainty requirements

## Abstract

International scientific standards organizations created standards on evaluating uncertainty in the early 1990s. Although scientists from many fields use these standards, they are not consistently implemented in the remote sensing community, where traditional error analysis framework persists. For a satellite instrument under development, this can create confusion in showing whether requirements are met. We aim to create a methodology for translating requirements from the error analysis framework to the modern uncertainty approach using the product level requirements of the Advanced Baseline Imager (ABI) that will fly on the Geostationary Operational Environmental Satellite R-Series (GOES-R). In this paper we prescribe a method to combine several measurement performance requirements, written using a traditional error analysis framework, into a single specification using the propagation of uncertainties formula. By using this approach, scientists can communicate requirements in a consistent uncertainty framework leading to uniform interpretation throughout the development and operation of any satellite instrument.

## 1. Introduction

Results of measurements are incomplete unless they are accompanied by a statement of associated uncertainty. Traceability to the SI units of measurement and inter-comparison of measurements require properly quantified measurement uncertainties. Various scientific communities have adopted different ways of expressing uncertainty, sometimes using different vocabulary. This has resulted in confusion in communicating experimental results and their interpretations. Scientists and statisticians in the metrology community have grappled with this problem and—through the participation of National Measurement Institutes, the International Organization for Standardization (ISO) and other international scientific standards organizations—established the Guide to the Expression of Uncertainty in Measurement (GUM) in the early 90s [1]. It has been adopted by the Bureau International des Poids et Measures (BIPM), the international body of Metrology in Paris that guides standardization in terms of SI units for all nations of the world. Also BIPM published the Vocabulary in Metrology (VIM) [2]. The GUM and VIM are being implemented across all scientific disciplines through an accreditation process for metrology laboratories in the government, industry and academia by various international scientific and technical standards organizations including the ISO and the OIML (International Organization for Legal Metrology). In the remote sensing community, there is a legacy of stating requirements following traditional analysis concepts.

This short paper addresses the error analysis based concepts and translates them into the terminology and methodology of the GUM. This translation will allow instrument vendors and calibration scientists following the new ISO standards of VIM and GUM to relate with the requirements expressed in traditional error analysis concepts. Such an understanding could pave the way for easier implementation of the GUM in the remote sensing metrology community. We review the current practice in remote sensing requirements using the Advanced Baseline Imager (ABI) that will fly on the Geostationary Operational Environmental Satellite R-Series (GOES-R) as an example. This multispectral imager covers from the visible to long-infrared wavelengths with higher spatial, spectral, and temporal resolution than previous GOES Earth-observing instruments [3]. The radiometric requirements (in the Mission Requirements Document [MRD] [4]) were written using an error analysis terminology, so we propose a translation commensurate with VIM and GUM.

## 2. Measurement Uncertainty as Defined by the GUM

The GUM and VIM have become indispensable tools for unambiguous world-wide communication of measurement results in science and technology. The GUM is based on the concept that while the error in a measurement is unknowable, uncertainty in the state-of-knowledge can be quantified. Measurement uncertainty is the dispersion of values that could reasonably be attributed to the measurand (quantity intended to be measured). In most cases the result of measurement *y* for a measurand is determined from the values of *N* other quantities *x*_1_,…, *x_N_* through a specified model of measurement *y* = *f* (*x*_1_,…, *x_N_*). The basic expression of measurement uncertainty in the GUM is standard uncertainty, which is uncertainty expressed as a standard deviation. The standard uncertainties *u*(*x*_1_),…,*u*(*x_N_*) associated with the input quantity values *x*_1_,…, *x_N_* are combined to determine the combined standard uncertainty *u_c_*(*y*) using the propagation of uncertainties formula given below:

(1) $$u_{c}^{2}\left(y\right)=\sum_{i=1}^{N}c_{i}^{2}u^{2}\left(x_{i}\right)+2\sum_{i=1}^{N-1}\sum_{j=i+1}^{N}c_{i}c_{j}u\left(x_{i}\right)u\left(x_{j}\right)r\left(x_{i},x_{j}\right)$$

where *c*_1_,…,*c_N_* referred to as sensitivity coefficients are partial derivatives of the model *y* = *f* (*x*_1_,…,*x_N_*) with respect to the input quantities and *r*(*x_i_*, *x_j_*) are correlation coefficients between input quantities [1]. When the input quantities are uncorrelated, the second term in the propagation of uncertainty formula drops out. The pair (*y*,*u_c_* (*y*)) is a complete result of measurement in the GUM. However in certain applications the result of measurement is expressed as an interval. So GUM introduced the concept of expanded standard uncertainty *U* = *ku_c_* (*y*), where *k* is a multiple called coverage factor, which enables expressing uncertainty as the interval (*y* ± *U*) = (*y* ± *ku_c_*(*y*)). The coverage factor is usually *k* = 1, 2, or 3.

## 3. Current Practice

Previous to the establishment of the GUM, measurement uncertainties were frequently expressed in terms of error relative to some true value which is unknown and unknowable. For example, the MRD for GOES-R gives requirements in these terms, for instance in defining “product measurement accuracy” and “product measurement precision” [4]. Product measurement accuracy is defined for non-categorical products as the systematic difference or bias between the derived parameter and ground truth. Product measurement precision for non-categorical products is defined as the one-sigma standard deviation of the differences between the derived parameters and ground truth (over the same population of data used to compute the product measurement accuracy). These definitions refer to an inherently unknowable truth. If these requirements could be reframed in terms of measurement uncertainty as described in the GUM, they could be stated in a framework that is consistent with the international standards for expressing uncertainty in science, technology and commerce. In the next section, we illustrate making such a transformation. More details on the relationship between concepts of error analysis and the modern view of uncertainty based on the GUM are discussed in reference [5].

## 4. Translation to GUM Methodology

We illustrate the method for transforming these requirements using the accuracy and precision requirements introduced above by first trying to understand their intent. We make reasonable assumptions about the probability distributions that govern the implied contributions of uncertainty from the requirements and combine them according to the GUM framework.

**Fig. 1:**
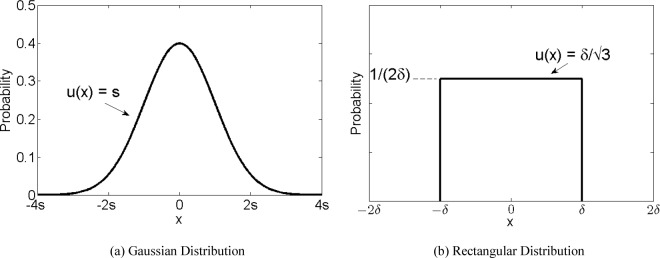
The two types of distribution used to describe the Advanced Baseline Imager requirements. (a) A Gaussian distribution (shown with standard uncertainty *u*(*x*) = *s*) is applied to precision and short-term repeatability requirements, and (b) a rectangular distribution (shown with $u\left(x\right)=\delta /\sqrt{3}$) is applied to accuracy and long-term drift requirements.

We interpret accuracy and precision requirement as uncertainty coming from two independent sources: bias and imprecision of the measurements. The requirement on bias is an upper bound so it is reasonable to assume that it represents the half-width of a rectangular distribution (Fig. 1b). On the other hand, the definition of precision informs the probability distribution; the intent of the requirement is to address the random effects that give rise to a standard deviation among a set of measurements, which is a signature of a Gaussian distribution (Fig. 1a). We can combine the uncertainties due to the bias and imprecision using the propagation of uncertainties formula stated above. This method transforms the accuracy/precision requirements into a single combined uncertainty requirement. In the pre-GUM framework this uncertainty is often referred to as “accuracy” or as “bias” alone leading to confusion [6,7]. VIM and GUM avoid such confusion as they clearly define the usage of uncertainty and its components arising from random effects and for corrections applied for systematic effects.

For the product measurement requirements of the reflective solar channels of ABI, the MRD gives the requirements in terms of accuracy, short term repeatability, and long term drift [4]. We treat this accuracy by considering the standard deviation of a rectangular distribution with half-width *δ*. The standard uncertainty is then $u\left(x\right)=\delta /\sqrt{3}$ where *δ* = 5%. Similarly, for long-term drift the standard uncertainty is $u\left(x\right)=\delta_{d}/\sqrt{3}$, where *δ_d_* = 1.5%. Short term repeatability is analogous to precision, so we assign it a Gaussian distribution with a standard uncertainty of *s* = 0.2%. The three sources of uncertainty are combined by the propagation of uncertainties formula:

(2) $$u_{c}\left(y\right)=\sqrt{\frac{\delta_{d}^{2}}{3}+s^{2}+\frac{\delta^{2}}{3}}$$

This gives a combined standard uncertainty of *u_c_* (*y*) = 3.02 % (*k* = 1). Again, since the precision is given in reference to a one-sigma value, we consider the *k* =1 value as the uncertainty requirement.

This analysis can be extended directly to other requirements and other instruments. Vendors of satellite instruments must meet lower-level requirements, and like the MRD, they are frequently stated in an error analysis framework instead of the uncertainty framework based on the GUM. This leaves them with the difficult job of determining how to interpret the requirements. Using the approach described in this work, vendors can perform uncertainty analyses and then directly compare their results with requirements stated in terms of uncertainty.

## 5. Conclusion

This short paper illustrates a methodology for translating the requirements written in the error analysis concepts to the measurement uncertainty framework, which has been internationally recognized as the preferred standard approach while preserving the intent of the requirements. We describe an example from the Mission Requirements Document of ABI to illustrate the method. Requirements analyses are often done working in a framework that inherently generates ambiguity. We have attempted to remove this ambiguity by introducing a way of working in a common internationally-accepted framework. We hope this methodology will be accepted by the GOES-R and remote sensing community in order to improve understanding of satellite instrument performance throughout development and operation. We also hope that in future projects for space bound sensors, the uncertainty requirement statements could be written unambiguously by following the ISO GUM as discussed in this paper to avoid confusion during project implementation.
